# Comprehensive Analysis of REST/NRSF Gene in Glioma and Its ceRNA Network Identification

**DOI:** 10.3389/fmed.2021.739624

**Published:** 2021-11-11

**Authors:** Yulian Zhang, Qi Wang, Zai Wang, Chuanpeng Zhang, Xiaoli Xu, Jun Xu, Hongxiang Ren, Xu Shao, Xueke Zhen, Li Zhang, Yanbing Yu

**Affiliations:** ^1^Department of Neurosurgery, China-Japan Friendship Hospital, Beijing, China; ^2^Department of Neurosurgery, Peking University China-Japan Friendship School of Clinical Medicine, Beijing, China; ^3^Institute of Clinical Medical Sciences, China-Japan Friendship Hospital, Beijing, China; ^4^Department of Neurosurgery, Graduate School of Peking Union Medical College, Beijing, China

**Keywords:** REST (RE-1 silencing transcription factor), glioma, therapeutic target, ceRNA, experiment validation

## Abstract

We sought to clarify the clinical relationship between REST/NRSF expression and the prognosis of glioma and explore the REST-associated competitive endogenous RNA (ceRNA) network in glioma. We downloaded RNA-seq, miRNA-seq and correlated clinical data of 670 glioma patients from The Cancer Genome Atlas and analyzed the correlation between REST expression, clinical characteristics and prognosis. Differentially expressed genes (DEGs) were identified with DESeq2 and analyzed with Gene Ontology (GO) and the Kyoto Encyclopedia of Genes and Genomes (KEGG) using the Profiler package. Starbase was used to explore the regulatory interaction between REST and miRNAs or LncRNAs. The lncRNA-miRNA-REST ceRNA network was constructed with Cytoscape. RT-qPCR, WB, CCK8, wound-healing, and luciferase assays were performed to validate the ceRNA network. Results showed that REST expression was significantly higher in glioma patients than normal samples. Higher REST expression was significantly associated with worse overall survival, progression-free interval, and worse disease-specific survival in glioma patients. The DEGs of mRNA, miRNA, and lncRNA were identified, and GO and KEGG enrichment analyses were performed. Finally, REST-associated ceRNA networks, including NR2F2-AS1-miR129-REST and HOTAIRM1-miR137-REST, were experimentally validated. Thus, REST may be a prognostic biomarker and therapeutic target in glioma, and its regulatory network validated in this study may provide insights into glioma's molecular regulatory mechanisms.

## Introduction

Glioma is the most common primary intracranial tumor worldwide. Although surgery combined with chemotherapy and radiation can prolong the recurrence and survival time, the overall treatment for glioma remains unsatisfactory, and high recurrence rate and high mortality remain common (1, 2).

Repressor element 1 (RE-1) silencing transcription factor (REST), also known as neuron-restrictive silencer factor (NRSF), is an important negative neuronal transcriptional regulator and plays a pivotal role in the regulation of ion channel expression, synaptic plasticity, neurotransmitter receptors and the terminal differentiation of neurons (3–5). REST exerts its effects in brain development, neural differentiation, neurodegenerative diseases, aging and brain tumors (6, 7). REST often functions as an oncogene in tumors including small cell lung cancer (8), ovarian cancer (9) and oral squamous cell carcinoma (10). With regards to central nervous system (CNS) tumors, several studies have found that REST expression level is high in neuroblastoma (4, 11, 12), medulloblastoma (13) and glioblastoma (GBM) (14–17). Moreover, downregulation of REST inhibits the self-renewal potential and tumor-initiating capacity of GBM cells (15). While regulating thousands of target genes, REST itself is also involved in various regulatory mechanisms, including ubiquitination (18), proteasome degradation and nuclear translocation (19). REST has been considered an important therapeutic target for the treatment of glioma. Therefore, understanding REST function and how REST is regulated are especially important problem in molecular biological research.

However, the clinical relevance, gene function, and the regulatory mechanism of REST-associated ceRNA network remain unknown in glioma. In this study, we analyzed the clinical relevance of REST expression, identified REST-associated differential expression genes (DEGs) and performed functional enrichment analysis, and explored the REST-associated ceRNA regulatory network in glioma with its associated differentially expressed (DE)-RNAs, based on The Cancer Genome Atlas (TCGA) database. Our findings may provide new insights into the clinical prognostic value and regulatory mechanisms of REST in glioma.

## Methods

### Data Acquisition

All microarray expression data, including RNA-seq and miRNA-seq, and their correlated clinical data were download from The Cancer Genome Atlas (20) (TCGA) database (LGG and GBM datasets). After invalid information was discarded, 670 RNA-seq data of glioma patients with clinical information were finally obtained. RNA-seq data of level 3 HTSeq-FPKM were transformed into TPM (transcripts per million reads) format for subsequent analysis. The clinical data which were “Unavailable” or “Unknown” were considered as missing values. The prognosis data, such as overall survival (OS), progression-free interval (PFI) and disease-specific survival (DSS), were obtained from the study of Liu (21). The RNA-seq data of normal brain samples from standardized Genotype-Tissue Expression (GTEx) (22) were considered as normal controls and were obtained from the Xena (23) database (https://xenabrowser.net/).

### Identification of DEGs

The data were divided into high and low REST expression groups, and the expression profile data of the top 30% (0–30%) and the bottom 30% (70–100%) were extracted for subsequent analysis. The DESeq2 package (24) was used to identify the DEGs of glioma. HTSeq counts were used for the analysis of DE-mRNAs and DE-lncRNAs, while miRNA-seq counts were used for the analysis of DE-miRNAs. |logFC| > 1.5 and adjusted *p* < 0.05 was set as the cut-off criteria for DEGs, and the results were visualized with volcano plot and heatmap.

### Functional Enrichment Analysis

The Cluster Profiler package (25) was used for functional enrichment analysis. To explore the possible functions, annotations of the REST-related DEGs, GO enrichment analysis and KEGG pathway enrichment analysis were conducted and visualized.

### Human Protein Atlas

Differences in REST protein expression between glioma and normal brain tissues were examined by immunohistochemistry (IHC) images from the HPA database (26). The staining intensity evaluation was determined from the HPA (https://www.proteinatlas.org).

### ceRNA Network Construction

Starbase (27) (http://starbase.sysu.edu.cn/) was used to explore the regulatory interaction between REST and miRNA or lncRNA, and the lncRNA-miRNA-REST ceRNA network was constructed with Cytoscape software (28).

### Cell Lines and Culture

Human cell lines U251, T98G, and 293T were purchased from ATCC and cultured in DMEM medium (Invitrogen, Thermo Fisher Scientific, USA). Culture media contained 10% fetal bovine serum (FBS) (Gibco) plus penicillin Streptomycin (Gibco), GlutaMAX(Gibco), and MEM non-Essential Amino Acids (MEM-NEAA) (Gibco). The above cells were all incubated in an incubator at 37°C with 5% CO2.

### Cell Transfection

The mimics of miRNAs and siRNA were obtained from GenePharma Co. Ltd (Shanghai, China). The primers of all the genes were synthesized from TsingKe Biological Technology (Beijing, China). All the sequences were displayed in Supplementary Table 1. Transfection of miRNAs and siRNA was performed using Lipofectamine 3000 (Invitrogen, Carlsbad, CA, USA) when the cell density reached approximately 50%-70%(usually 24 h after inoculation) following instruction of manufacture, transfection of plasmid was performed using Hieff TransTM Liposomal Transfection Reagent (YEASEN, Shanghai, China), according to the manufacturer's instructions. The concentrations of the siRNA and miRNA used in transfection was 5 pmol/well for 96-well-plate (40 pmol/well for 12-well-plate and 100 pmol/well for 6-well-plate, respectively) following the manufacturer's instructions (GenePharma Co. Ltd, Shanghai, China).

### RNA Extraction, RT-qPCR, and Western Blot

The methods of RNA extraction and real-time RT-PCR analysis were performed as previously described (29). Briefly, total RNA was extracted and converted into complementary DNA (cDNA). Quantitative PCR (qPCR) was performed using QuantiNova SYBR Green PCR Kit (QIAGEN, Hilden, Germany). Quantifications were normalized and calculated by the 2^−ΔΔCt^ method.

Western blot was performed as previously (29). Briefly, total protein was quantified using bicinchoninic acid (BCA) method and separated using 10% SDS-PAGE. Then the gels were transferred to PVDF membranes. After blocked with 5% skim milk in TBST, the membranes were incubated with the primary antibodies: GAPDH (1:6000; Proteintech, USA) and REST (1:2000; Proteintech, USA) overnight at 4°C. After washing with TBST, the membranes were reacted with the secondary antibody. Finally, enhanced chemiluminescence (ECL) kit (MD Millipore, Germany) was used to measure the signals in the Bio-Rad ChemiDocXRS Imaging system (Bio-Rad, Hercules, CA, USA) and calculated with Image J software.

### CCK8 Assay

The proliferative capacity of glioma cells was determined using the CCK8 kit (DOJINDO, Japan). After cell transfection, log phase grown cells were implanted in 96-well-plates with 3,000 cells per well. At 0, 24, 48, and 72 h post-transfection, 10 μl CCK-8 reaction solution was added to each well. The plates were then placed back in the incubator for 1 h. Finally, the absorbance of each well was measured at 450 nm using a multifunctional enzyme marker.

### Wound Healing Assay

After cell transfection, cells were digested and resuspended with serum-free DMEM culture medium, inoculated into 6-well-plates at a cell count of 150,000 per well, and cell transfection was performed at 24 h. When the cells were grown to fusion (usually 48 h after inoculation), a 200 ul pipette tip was used to make a vertical “wound” in the cell layer. This was followed by incubation with DMEM containing 2% FBS to eliminate the effect of proliferation on the migratory phenotype. Scratch pictures were collected at 0, 24, 48, and 96 h (until the scratch healed in the experimental or control group). Image J software was applied to calculate the scratch healing area. Scratch healing ratio of X h = (0 h scratch area–X h scratch area)/0 h scratch area.

### Luciferase Assay

The methods of luciferase assay were performed as previously described (29). Direct target sites were predicted based on TargetScan (http://www.targetscan.org/vert_72/) and starBase v2.0 (http://starbase.sysu.edu.cn/) (27). The luciferase plasmid was constructed, and the 3′-UTR sequences of REST and lncRNAs were cloned into psiCHECK-2 promoter vector (Promega) by Ruibio Biotech (Beijing, China).

### Statistical Analysis

All statistical analyses were performed with R (v3.6.2) or GraphPad Prism 8.0 (GraphPad Inc., California, CA, USA). Wilcoxon rank sum test was used to analyze the expression of glioma and normal samples. Receiver operating characteristic (ROC) and the area under curve (AUC) were used to test the diagnostic value of REST expression for glioma. Kruskal–Wallis test and Wilcoxon signed-rank test were used to analyze the correlation between REST expression and clinical characteristics. Spearman rank correlation was used to analyze the correlation between REST expression and hub genes in ceRNA network. Cox regression analysis or Kaplan-Meier analysis were used to evaluate the correlation between REST expression and clinical prognosis. In Cox regression analysis, variables with p < 0.1 in univariate Cox regression were included in multivariate Cox regression analysis. Spearman correlation test was used to analyze the correlation of gene expression. *P* < 0.05 were considered statistically significant.

## Results

### REST Was Significantly Up-Regulated in Glioma

**Figure 1:**
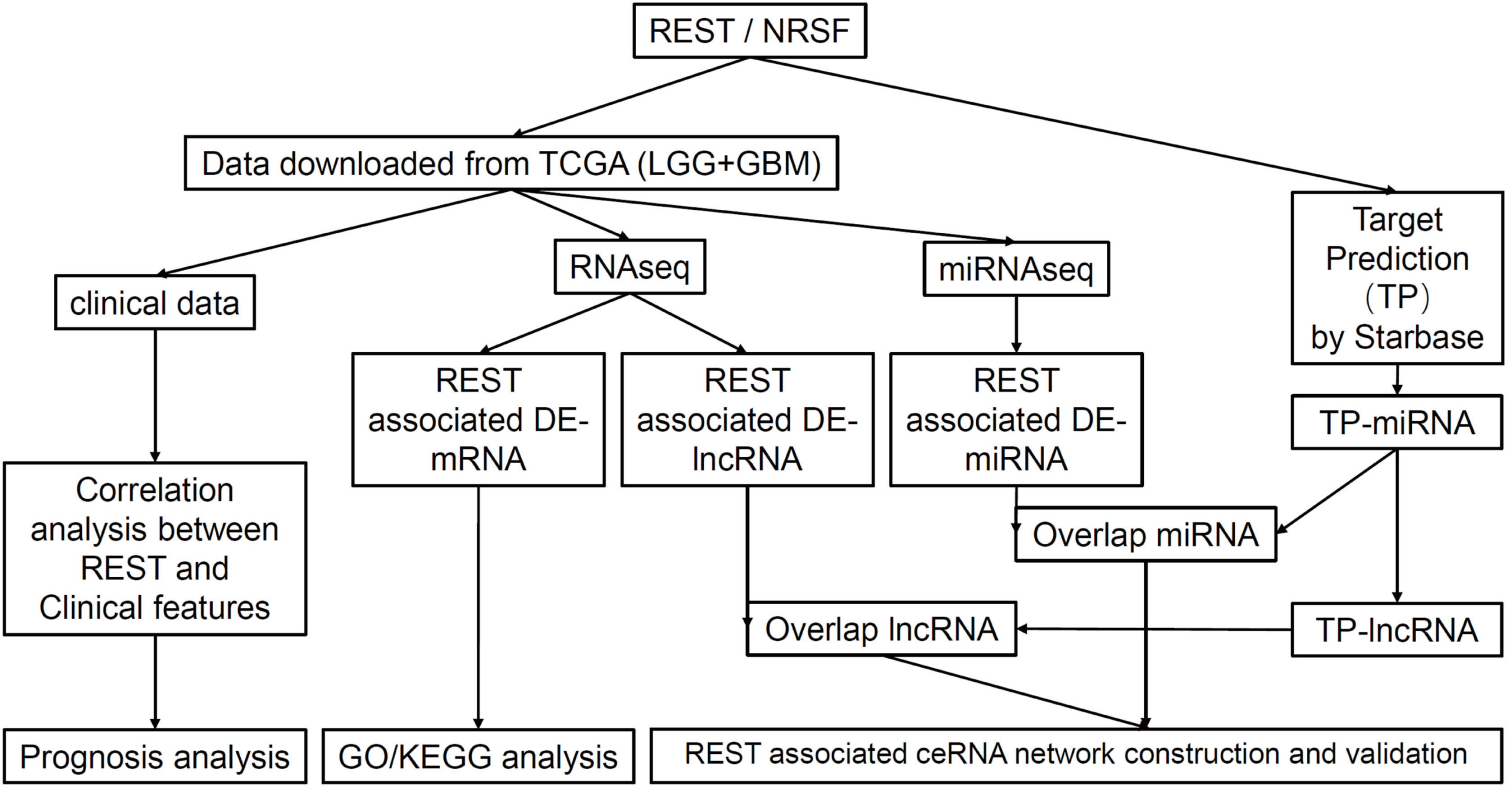
Flow chart of the study.

**Figure 2:**
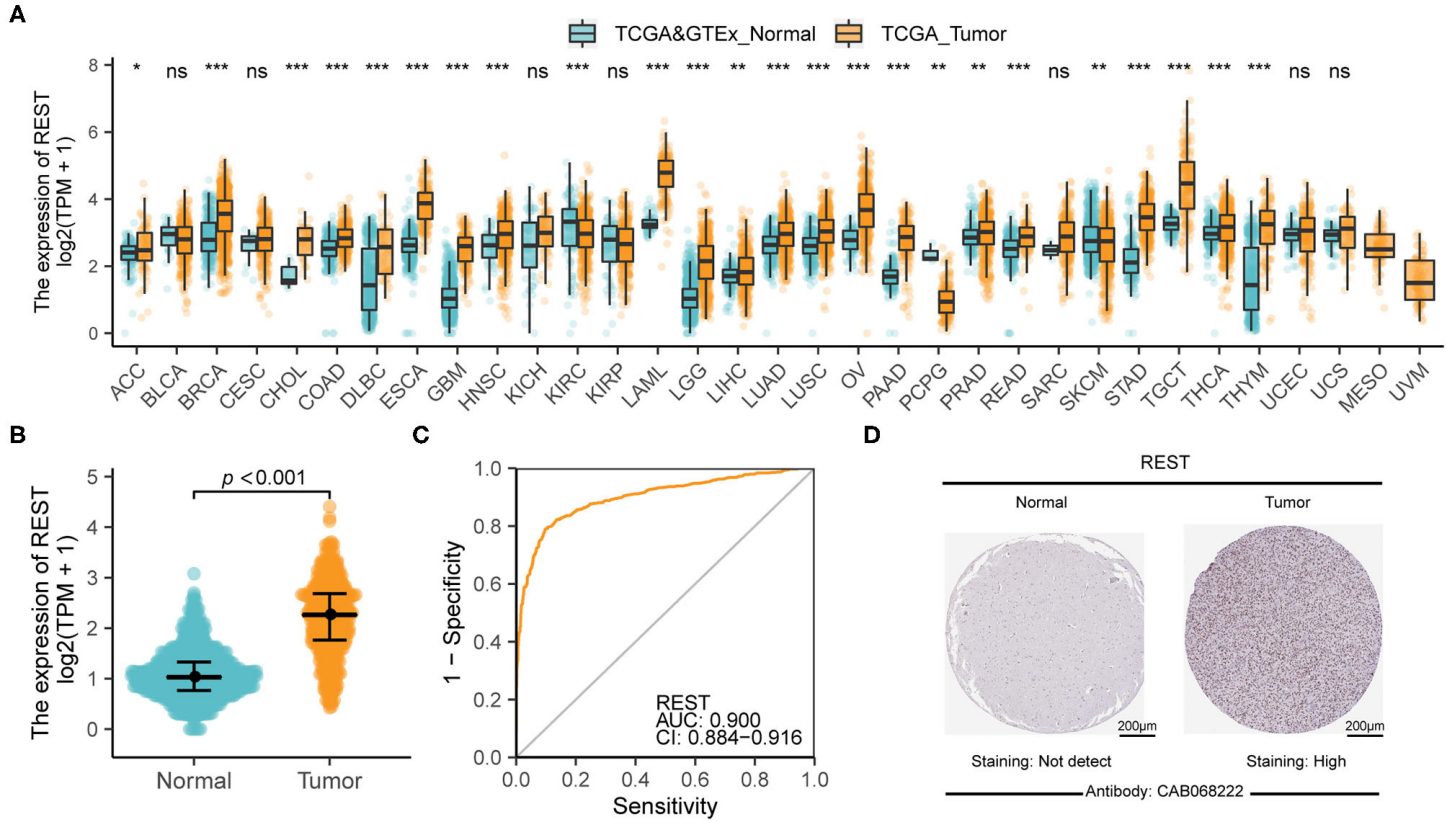
REST was significantly up-regulated in glioma. **(A)** The difference of REST expression in pan-cancer and adjacent tissues. **(B)** REST differential expression analysis between glioma samples in TCGA database and normal brain samples from TCGA and GTEx databases. **(C)** The diagnostic value of REST in glioma. **(D)** The representative immunohistochemical images of REST in the Human Protein Atlas database of glioma and normal tissues. All data are shown as mean ± SD, *P < 0.05, **P < 0.01, and ***P < 0.001.

The flow chart of our study is shown in Figure 1. To analyze the difference in REST expression between gliomas and normal tissues, we used the TCGA database combined with GTEX data to analyze the gene expression level of REST in pan-cancer and adjacent tissues. The results showed that the expression of REST was significantly up-regulated in most tumors compared to normal tissues, such as esophageal carcinoma, acute myeloid leukemia (LAML) and testicular germ cell tumors (TGCT) (all *p* < 0.001, Figure 2A). Furthermore, we observed that REST expression level in glioma (LGG + GBM) was significantly higher than that in normal samples (Figure 2B). The ROC curve was used to analyze the diagnostic value of REST. The AUC was 0.9, which indicates that REST is a potential diagnostic biomarker (Figure 2C). In addition, we explored the protein expression of REST in glioma and normal samples and examined the immunohistochemical results of REST protein using the HPA (Supplementary Figure 1). The representative results showed that REST was not expressed in normal brain tissue, whereas high expression of REST was observed in glioma tissue (Figure 2D).

### High Expression of REST Was Associated With Worse Characteristics

**Figure 3:**
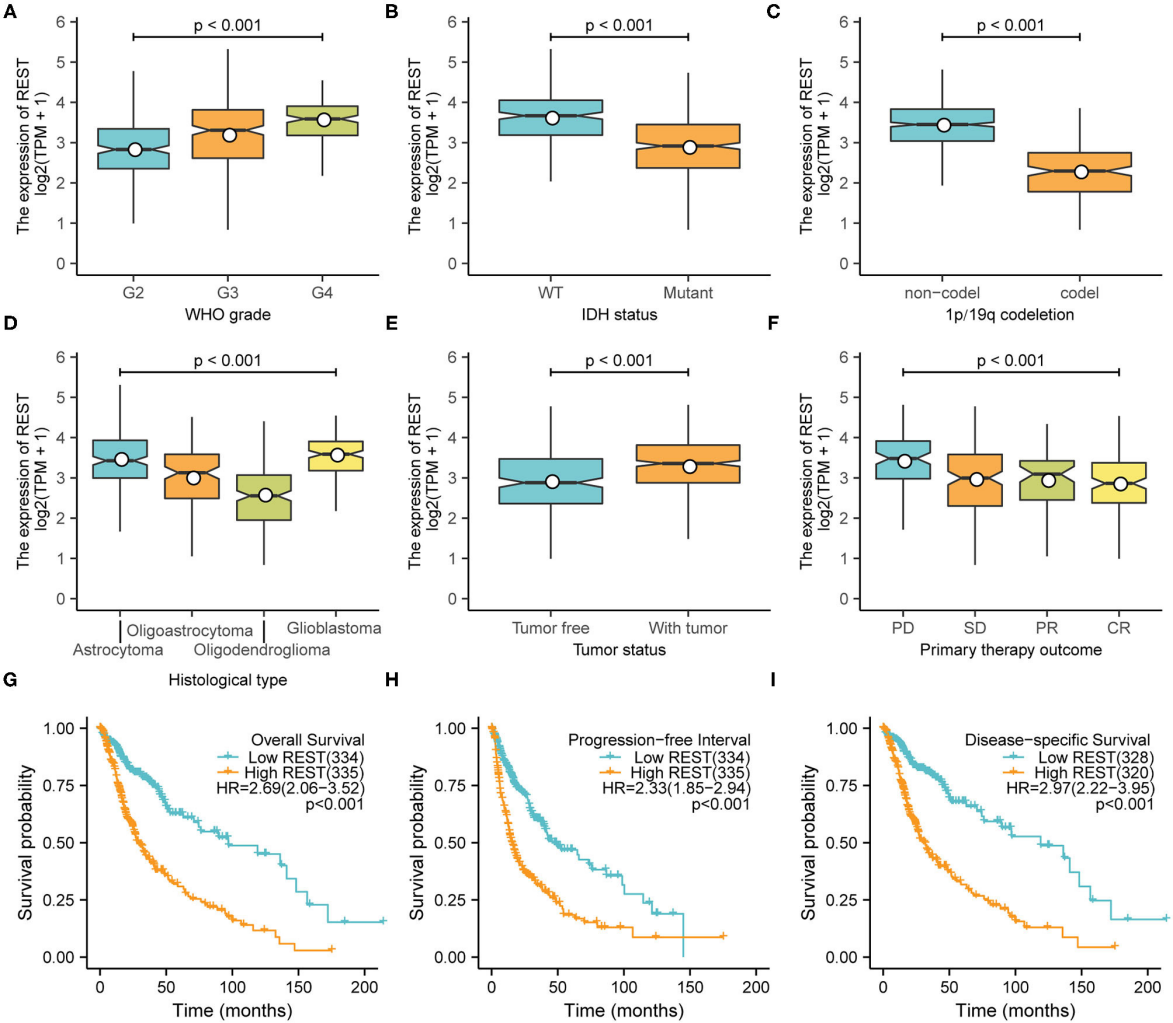
High expression of REST was associated with worse characteristics. The correlation between REST expression and WHO stage **(A)**, IDH status **(B)**, 1p/19q codeletion **(C)**, histological type **(D)**, tumor status **(E)**, and primary treatment outcome **(F)**. Prognostic analysis of REST expression and overall survival **(G)**, progression-free interval **(H)**, and disease-specific survival **(I)**. Data were analyzed using Kruskal–Wallis test **(A,D,F)**, Wilcoxon signed-rank test **(B,C,E)**, and Kaplan–Meier analysis **(G–I)**.

The Kruskal–Wallis test and Wilcoxon signed-rank test were used to analyze the correlation between REST expression and clinical characteristics of glioma. We found that higher REST expression level positively correlated with higher WHO grade (*p* < 0.001, Figure 3A), IDH status-WT (*p* < 0.001, Figure 3B), non-1p/19q codeletion (*p* < 0.001, Figure 3C), histological type-GBM (*p* < 0.001, Figure 3D), tumor status -with tumor (*p* < 0.001, Figure 3E) and primary therapy outcome-PD (*p* < 0.001, Figure 3F). Consistent results were obtained from chi-square test (Table 1). Logistic regression analysis also suggested similar results. The results showed that REST expression was significantly correlated with 1p/19q codeletion (non-codeletion) (OR = 28.73, *p* < 0.001), IDH status (WT) (OR = 4.60, *p* < 0.001), WHO grade (G3–4) (OR = 3.84, *p* < 0.001), histological type (GBM) (OR = 3.44, *p* < 0.001), tumor status (with tumor) (OR = 2.5, *p* < 0.001), primary therapy outcome (SD + PD) (OR = 2.09, *p* < 0.001) and Karnofsky score (≤80) (OR = 2.01, *p* < 0.001, Table 2). These results suggest that REST expression is closely related to worse clinical characteristics.

**Table 1:** Correlation between REST expression and clinical characteristics in glioma patients.

**Characters**	**Level**	**Low expression of REST**	**High expression of REST**	***p***
*n*		335	335	
OS event (%)	Alive	257 (76.7)	159 (47.5)	<0.001
	Dead	78 (23.3)	176 (52.5)	
WHO grade (%)	G2	175 (52.2)	73 (21.9)	<0.001
	G3	116 (34.6)	145 (43.4)	
	G4	44 (13.1)	116 (34.7)	
IDH status (%)	WT	64 (19.3)	173 (52.4)	<0.001
	Mutant	267 (80.7)	157 (47.6)	
1p/19q codeletion	Non-codel	176 (52.7)	320 (97.0)	<0.001
	codel	158 (47.3)	10 (3.0)	
Histological type (%)	Astrocytoma	70 (20.9)	122 (36.4)	<0.001
	Oligoastrocytoma	70 (20.9)	58 (17.3)	
	Oligodendroglioma	151 (45.1)	39 (11.6)	
	Glioblastoma	44 (13.1)	116 (34.6)	
Tumor status (%)	Tumor free	140 (45.6)	79 (25.2)	<0.001
	With tumor	167 (54.4)	234 (74.8)	
Primary therapy outcome (%)	PD	36 (14.8)	67 (33.5)	<0.001
	SD	80 (32.8)	64 (32.0)	
	PR	38 (15.6)	24 (12.0)	
	CR	90 (36.9)	45 (22.5)	
Age (%)	≤ 45	176 (52.5)	155 (46.3)	0.122
	>45	159 (47.5)	180 (53.7)	
Gender (%)	Female	147 (43.9)	137 (40.9)	0.482
	Male	188 (56.1)	198 (59.1)	
Karnofsky score (%)	≤ 80	70 (37.6)	126 (54.8)	0.001
	>80	116 (62.4)	104 (45.2)	

**Table 2:** Logistic regression of clinical characteristics based on REST expression.

**Characteristics**	**Total (*N*)**	**Odds ratio in REST expression**	***P*-value**
WHO grade (G3–4 vs. G2)	669	3.84 (2.78–5.56)	<0.001
IDH status (WT vs. mutant)	661	4.60 (3.26–6.54)	<0.001
1p/19q codeletion (non-codel vs. codel)	664	28.73 (15.52–59.50)	<0.001
Histological type (Glioblastoma vs. low grade glioma)	670	3.44 (2.38–5.26)	<0.001
Tumor status (With tumor vs. tumor free)	620	2.5 (1.78–3.44)	<0.001
Primary therapy outcome (SD-PD vs. PR-CR)	444	2.09 (1.43–3.09)	<0.001
Age (≤45 vs. >45)	670	0.78 (0.57–1.05)	0.105
Gender (Female vs. Male)	670	0.88 (0.65–1.20)	0.434
Karnofsky score (≤80 vs. >80)	416	2.01 (1.36–2.99)	<0.001

### High Expression of REST Was Associated With Poor Clinical Outcomes

To explore the relationship between REST expression and prognosis of glioma patients, we analyzed the correlation of REST expression with OS, PFI and DSS. The results showed that higher REST expression was associated with worse OS (HR = 2.69; 95% CI, 2.06–3.52; Figure 3G), worse PFI (HR = 2.33; 95% CI, 1.85–2.94; *p* < 0.001; Figure 3H) and worse DSS (HR = 2.97; 95% CI, 2.22–3.95; *p* < 0.001; Figure 3I). Univariate Cox regression analysis of OS showed that WHO grade (G3–4), IDH status (WT), 1p/19q codeletion (non-codeletion), histological type (GBM), tumor status (with tumor), primary therapy outcome (SD + PD), age (>45), Karnofsky score (≤80) and high REST expression level was associated with poor OS (all *p* < 0.001, Table 3). Further multivariate Cox regression analysis suggested that high REST expression level were an marginally independent prognostic marker of OS.

**Table 3:** The overall survival (OS) univariate and multivariate cox proportional risk analysis.

**Characteristics**	**Univariate analysis**		**Multivariate analysis**	
	**Hazard ratio (95% CI)**	***P*-value**	**Hazard ratio (95% CI)**	***P*-value**
WHO grade (G2 vs. G3–4)	5.874 (4.116–8.382)	<0.001	1.602 (0.872–2.944)	0.129
IDH status (Mutant vs. WT)	9.803 (7.407–12.987)	<0.001	3.247 (1.821–5.780)	<0.001
1p/19q codeletion (codel v. non-codel)	4.629 (2.959–7.246)	<0.001	1.182 (0.521–2.681)	0.689
Histological type (Low grade glioma vs. glioblastoma)	9.045 (6.905–11.85)	<0.001	1.964 (0.442–8.718)	0.375
Tumor status (Tumor free vs. with tumor)	16.342 (7.701–34.678)	<0.001	5.981 (1.421–25.169)	0.015
Primary therapy outcome (PR-CR vs. SD-PD)	4.901 (2.740–8.771)	<0.001	2.096 (1.01–4.348)	0.047
Age (≤45 vs. >45)	4.49 (3.365–5.991)	<0.001	1.89 (1.07–3.34)	0.028
Gender (Female vs. male)	1.23 (0.955–1.585)	0.109		
Karnofsky score (>80 vs. ≤ 80)	3.921 (2.882–5.319)	<0.001	1.239 (0.734–2.092)	0.422
REST (Low vs. high)	2.689 (2.055–3.518)	<0.001	1.913 (0.998–3.668)	0.051

### REST-Associated DE-mRNA and GO/KEGG Analysis

**Figure 4:**
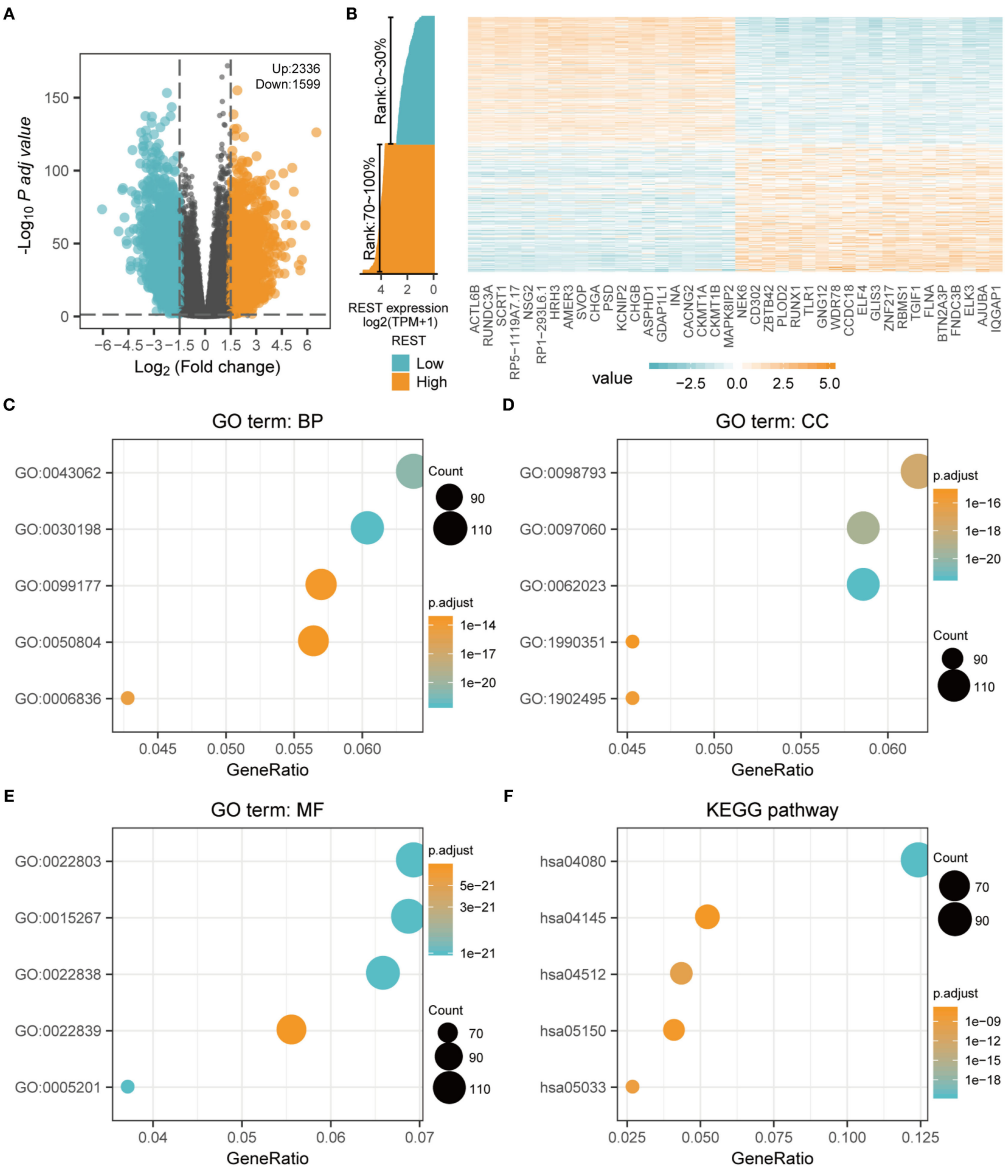
REST-associated differential expression gene analysis (DEGs) and GO/KEGG enrichment analysis. **(A)** The volcanic map showing the DE-mRNA of the high (top 30%) and low (bottom 30%) REST expression profiles. **(B)** The heat map showing the top 20 up-regulated and the top 20 down-regulated REST-associated DE-mRNA. **(C–F)** Functional enrichment analysis of REST associated DE-mRNA. The biological process **(C)**, cellular component **(D)**, molecular function **(E)**, and KEGG pathway **(F)** were analyzed.

To further explore the biological function of REST in glioma, we grouped the expression profiles according to the level of REST expression. We analyzed differences in whole-gene expression profile between samples with REST expression in the top 30% (0–30%) and those in the bottom 30% (70–100%), and found that 2,336 up-regulated genes and 1,599 down-regulated genes were significantly correlated with REST expression (|logFC| > 1.5 and adjusted *p* < 0.05, Figure 4A). The top 20 up-regulated and the top 20 down-regulated DE-mRNAs are shown in a heat map (Figure 4B). In addition, we conducted GO and KEGG enrichment analysis of the REST-associated DEGs. The results of GO analysis showed that changes in biological processes (BP) of REST-associated DEGs were mainly enriched in extracellular matrix (ECM) organization, extracellular structure organization and neurotransmitter transport (Figure 4C, Table 4), while changes in cellular components (CC) were mainly enriched in the collagen-containing ECM, synaptic membrane and pre-synapse (Figure 4D, Table 4). Changes in molecular function (MF) were mainly enriched in passive transmembrane transporter activity, channel activity and ECM structural constituents (Figure 4E, Table 4). KEGG pathway analysis revealed that the REST-associated DEGs were mainly enriched in neuroactive ligand-receptor interaction, ECM-receptor interaction, and nicotine addiction (Figure 4F, Table 4).

**Table 4:** GO/KEGG enrichment analysis of REST-associated DE-mRNAs.

**Category**	**ID**	**Description**	**GeneRatio**	***p*-value**	***p-*adjust**	***q*-value**
BP	GO:0030198	Extracellular matrix organization	107/1,781	7.3e-27	4.2e-23	3.2e-23
BP	GO:0043062	Extracellular structure organization	113/1,781	5.9e-25	1.7e-21	1.3e-21
BP	GO:0006836	Neurotransmitter transport	76/1,781	1.3e-18	2.4e-15	1.9e-15
BP	GO:0099177	Regulation of trans-synaptic signaling	101/1,781	1.8e-17	2.6e-14	2.0e-14
BP	GO:0050804	Modulation of chemical synaptic transmission	100/1,781	4.6e-17	5.2e-14	4.0e-14
CC	GO:0062023	Collagen-Containing extracellular matrix	111/1,902	6.7e-25	3.9e-22	2.9e-22
CC	GO:0097060	Synaptic membrane	111/1,902	1.6e-22	4.7e-20	3.6e-20
CC	GO:0098793	Pre-synapse	117/1,902	9.0e-21	1.8e-18	1.3e-18
CC	GO:1902495	transmembrane transporter complex	86/1,902	1.1e-18	1.7e-16	1.3e-16
CC	GO:1990351	Transporter complex	86/1,902	6.0e-18	7.0e-16	5.3e-16
MF	GO:0022803	Passive transmembrane transporter activity	121/1,758	1.2e-24	1.1e-21	8.9e-22
MF	GO:0015267	Channel activity	120/1,758	3.3e-24	1.1e-21	8.9e-22
MF	GO:0005201	Extracellular matrix structural constituent	65/1,758	3.3e-24	1.1e-21	8.9e-22
MF	GO:0022838	Substrate-Specific channel activity	115/1,758	4.6e-24	1.2e-21	9.3e-22
MF	GO:0022839	Ion gated channel activity	97/1,758	3.9e-23	7.9e-21	6.3e-21
KEGG	hsa04080	Neuroactive ligand-receptor interaction	98/798	9.1e-24	2.7e-21	2.1e-21
KEGG	hsa04512	ECM-receptor interaction	34/798	6.3e-13	9.2e-11	7.2e-11
KEGG	hsa05033	Nicotine addiction	21/798	1.5e-11	1.5e-09	1.1e-09
KEGG	hsa05150	*Staphylococcus aureus* infection	32/798	2.8e-10	2.1e-08	1.6e-08
KEGG	hsa04145	Phagosome	41/798	1.4e-09	8.4e-08	6.5e-08

### REST-Associated DE-lncRNA and DE-miRNA

**Figure 5:**
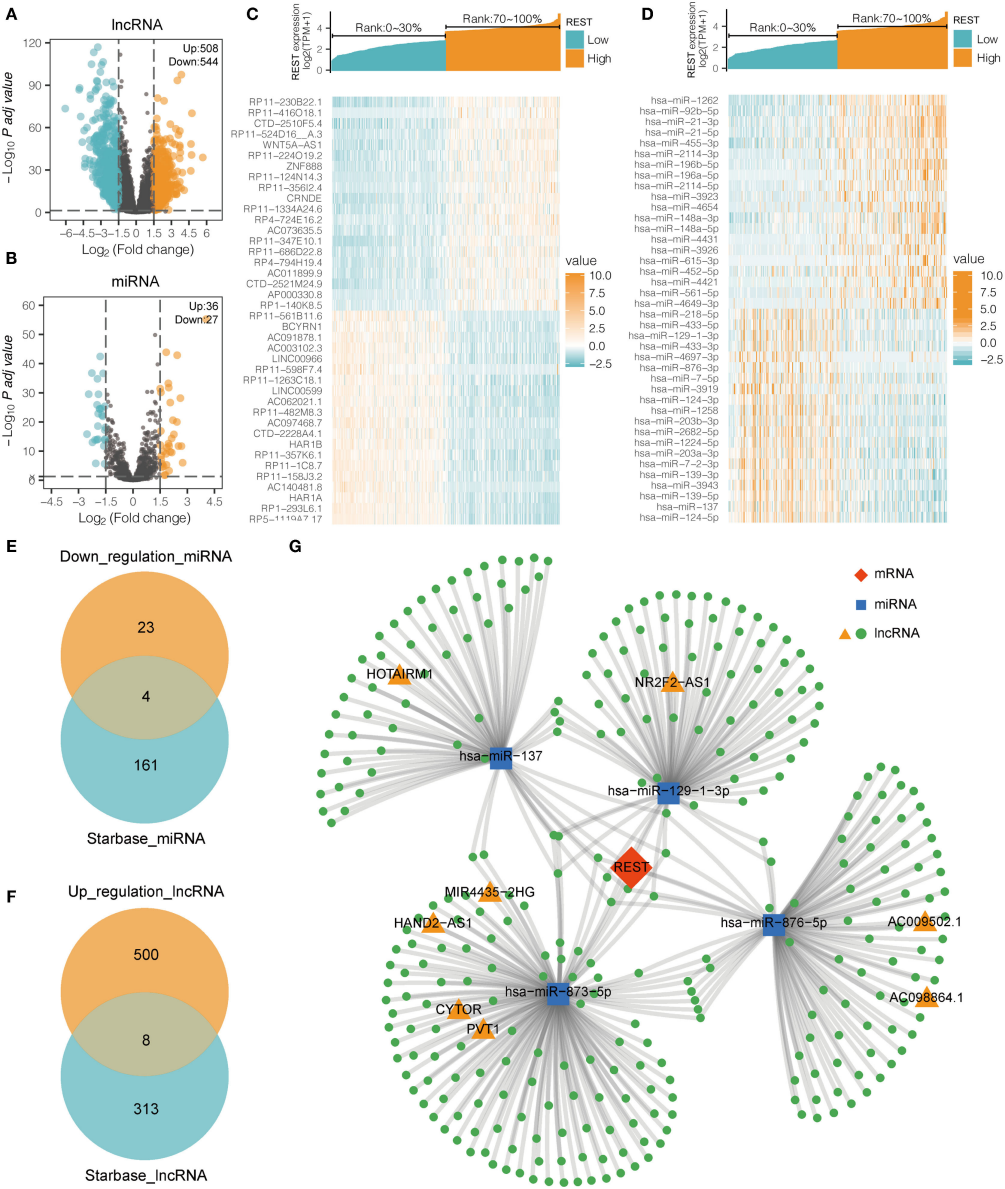
REST ceRNA network construction. **(A,B)** The volcanic map showing the DE-lncRNA **(A)** and DE-miRNA **(B)** of the high (top 30%) and low (bottom 30%) REST expression profiles. **(C,D)** The heat map showing the top 20 up-regulated and the top 20 down-regulated REST-associated DE-lncRNA **(C)** and DE-miRNA **(D)**. **(E)** Venn diagram illustrating the overlap between miRNAs that are associated with low expression of REST derived from TCGA database and miRNAs which target REST predicted by Starbase. **(F)** Venn diagram illustrating the overlap between lncRNAs that are associated with over expression of REST derived from TCGA database and the lncRNAs which target the overlap miRNAs predicted by Starbase. **(G)** LncRNA-miRNA-REST ceRNA network was constructed with predicted miRNAs and lncRNAs.

To study the lncRNA and miRNA associated with REST expression, we analyzed the DEGs of the lncRNA profile and miRNA profile based on the REST high-low expression group. In the lncRNA DEGs, there were 508 up-regulated lncRNAs and 544 down-regulated lncRNAs (|logFC| > 1.5 and adjusted *p* < 0.05, Figure 5A). In the miRNA DEGs, there were 36 up-regulated miRNAs and 27 down-regulated miRNAs (|logFC| > 1.5 and adjusted *p* < 0.05, Figure 5B). The top 20 up-regulated and the top 20 down-regulated DE-lncRNAs and DE-miRNAs are shown with a heat map (Figures 5C,D).

### REST-Associated ceRNA Network Construction

**Figure 6:**
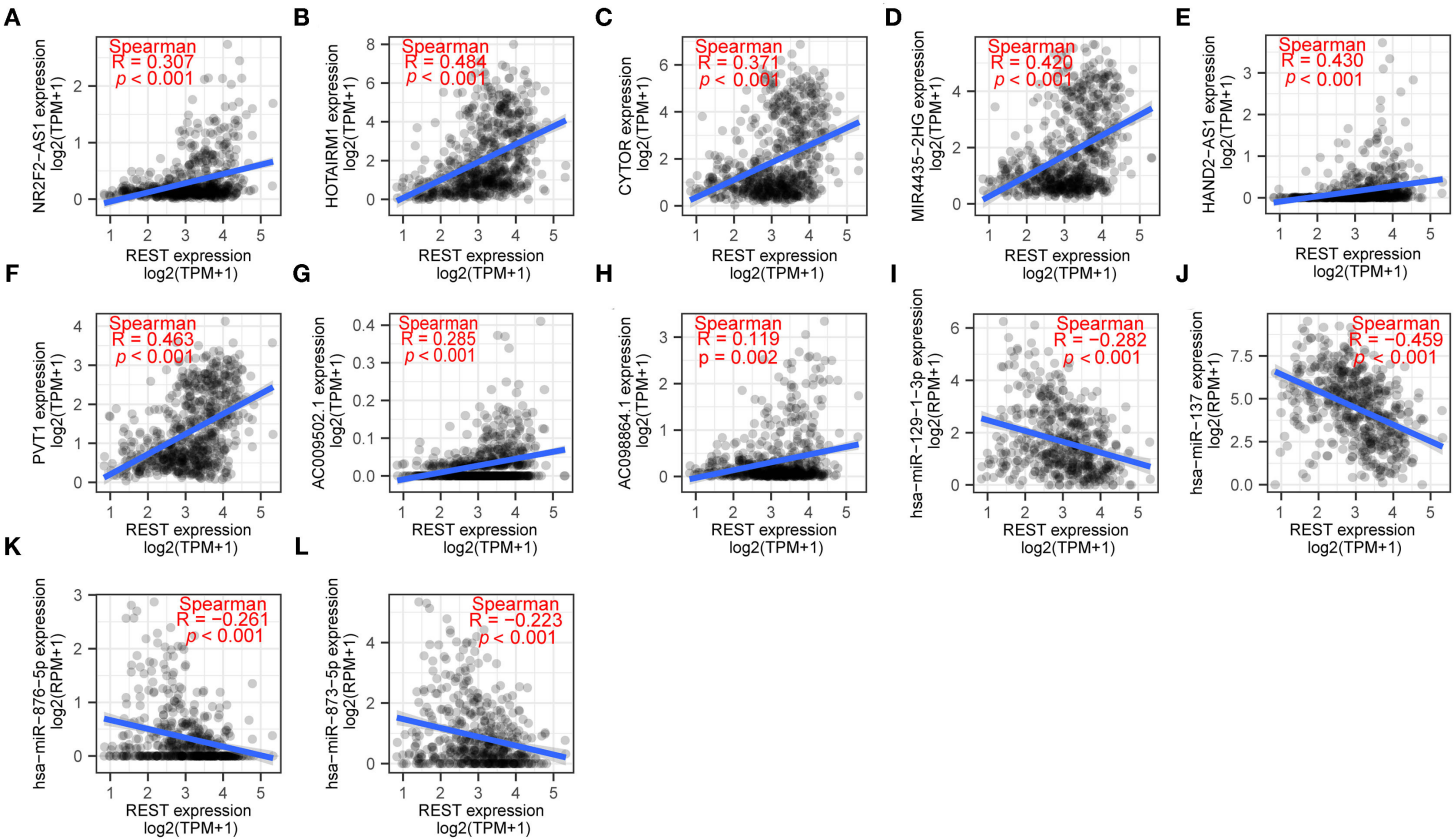
The correlation of REST and its hub genes of ceRNA network. The correlation between REST and NR2F2-AS1 **(A)**, HOTAIRM1 **(B)**, CYTOR **(C)**, MIR4435-2HG **(D)**, HAND2-AS1 **(E)**, PVT1 **(F)**, AC009502.1 **(G)**, AC0098864.1 **(H)**, hsa-miR-129-1-3p **(I)**, hsa-miR-137 **(J)**, hsa-miR-876-5p **(K)**, hsa-miR-873-5p **(L)**. Data were analyzed using Spearman rank correlation.

In recent years, the ceRNA hypothesis has suggested a new mechanism for RNA regulatory interactions (30). miRNA can silence genes by binding to the 3′UTR of mRNA, while ceRNA can competitively sponge miRNA to up-regulate the expression of its downstream genes. ceRNA and mRNA expression are positively correlated, while miRNA and mRNA expression are negatively correlated. To explore the REST-associated ceRNA regulatory network, we constructed a lncRNA-miRNA-REST network. Starbase (27) was used to predict miRNA that may target REST, which led to the discovery of 165 miRNAs; of which, 4 miRNAs were in the list of REST-associated down-regulated miRNAs (Figure 5E). These 4 miRNAs were used to predict 321 target lncRNAs; among which, 8 lncRNAs were in the list of REST-associated up-regulated lncRNAs (Figure 5F). We then constructed a lncRNA-miRNA-REST ceRNA network including predicted miRNAs and lncRNAs (Figure 5G). The correlation between miRNAs or lncRNAs obtained from the above analysis and REST expression was analyzed individually, and the results showed that the expression of NR2F2-AS1 (*R* = 0.307, *p* < 0.001), HOTAIRM1 (*R* = 0.484, *p* < 0.001), CYTOR (*R* = 0.371, *p* < 0.001), MIR4435-2HG (*R* = 0.420, *p* < 0.001), HAND2-AS1 (*R* = 0.430, *p* < 0.001), PVT1 (*R* = 0.463, *p* < 0.001), AC009502.1 (*R* = 0.285, p <0.001), and AC0098864.1 (*R* = 0.119, *p* = 0.002) were positively correlated with REST expression, while the expression of hsa-miR-137 (*R* = −0.459, *p* < 0.001), hsa-miR-129-1-3p (*R* = −0.282, *p* < 0.001), hsa-miR-876-5p (*R* = −0.261, *p* < 0.001), and hsa-miR-873-5p (*R* = −0.223, *p* < 0.001) were negatively correlated with REST expression (Figure 6).

### Experimental Validation

**Figure 7:**
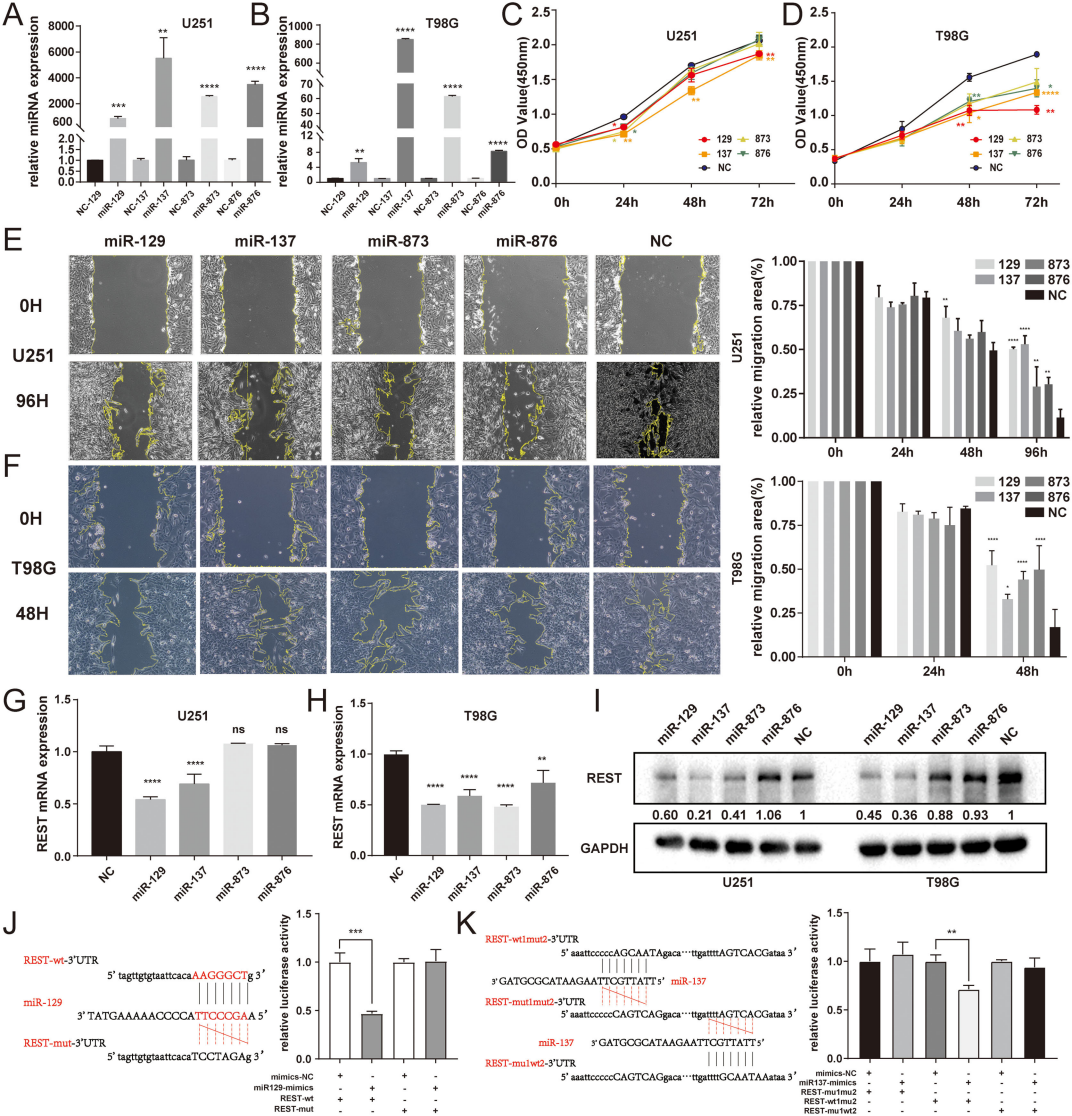
Experimental validation between REST and miRNAs of ceRNA. **(A,B)** The transfected expression of 4 miRNA-mimics in U251 and T98G cell lines. **(C,D)** Cell proliferation was determined by CCK8 assay. **(E,F)** Cell migration was determined by wound-healing assay. The expression levels of REST mRNA **(G,H)** and protein **(I)** were determined by qRT-PCR and Western Blot. **(J,K)** The binding site of miRNA and the 3′UTR of REST were predicted by Starbase and validated by luciferase assay. All data are shown as mean ± SD, *P < 0.05, **P < 0.01, ***P < 0.001, and ****P < 0.0001.

In order to determine the effectiveness of predicted miRNAs and its regulatory relationship with REST, we first overexpressed 4 miRNAs with miRNA-mimics and observed their own expression efficiency. The results showed that the level of miRNAs-mimics were significantly increased in U251 and T98G cell lines (Figures 7A,B). CCK8 assay was used to investigate the role of miRNAs in glioma cell growth and proliferation. The results showed that miR-129 and miR-137 can significantly inhibited the proliferation in glioma cell lines (Figures 7C,D). Wound healing assay was performed to evaluate the migration ability of miRNAs, and the results showed that all four miRNAs could significantly inhibit the migration of glioma cell lines (Figures 7E,F). Then, the silencing efficiency of REST mRNA (Figures 7G,H) and protein (Figure 7I) were determined by qRT-PCR and Western Blot. The results showed that only miR-129 and miR-137 can effectively regulate the expression levels of REST, at both mRNA and protein levels. Therefore, we selected miR-129 and miR-137, which can effectively regulate REST expression and have tumor inhibitory effects, for our further research. Then luciferase assays were performed to evaluate the direct binding of miR-129 or miR-137 with REST. The result showed that both miR-129 and miR-137 can directly bind to REST 3'UTR (Figures 7J,K).

**Figure 8:**
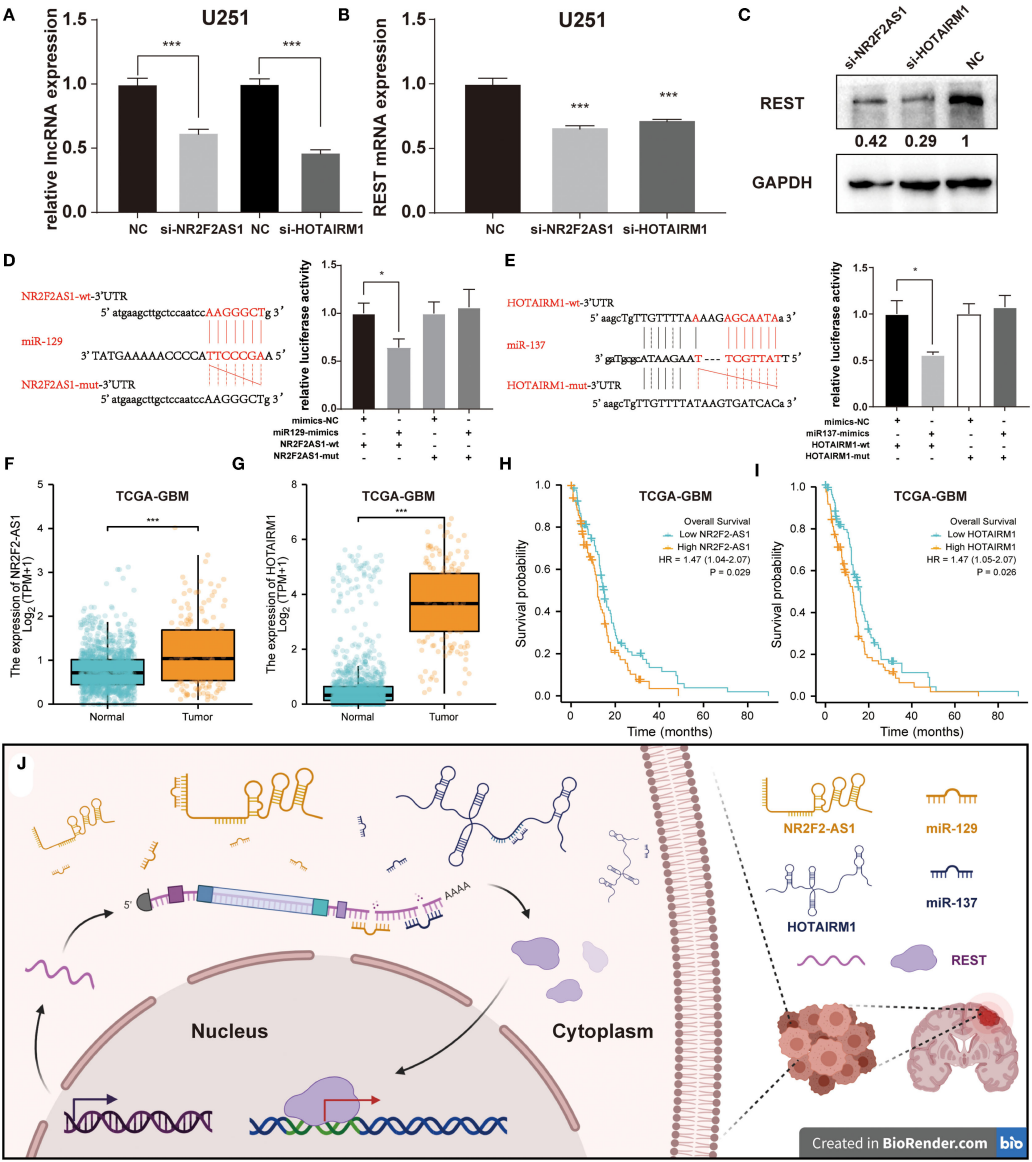
Experimental validation between REST, miRNA, and lncRNAs. **(A)** The relative LncRNA expression levels after transfection of si-lncNR2F2-AS1 and si-lncHOTAIRM1. The expression levels of REST mRNA **(B)** and protein **(C)** were determined by qPCR and Western Blot. **(D,E)** The binding site of miRNAs in relative lncRNAs were predicted by Starbase and validated by luciferase assay. **(F,G)** The differential expression analysis of lncRNA between glioma samples in TCGA-GBM database and normal brain samples from TCGA and GTEx databases. **(H,I)** Prognostic analysis of lncRNA expression and overall survival. **(J)** The mechanistic scheme of lncRNA-miRNA-REST in glioma. All data are shown as mean ± SD, *P < 0.05 and ***P < 0.001. The picture was created with BioRender.com.

Further, LncRNA NR2F2-AS1 and HOTAIRM1, which were predicted to be associated with miR-129 or miR-137, were selected for further experimental verification. First, we transfected U251 cells with siRNAs of LncRNAs to knockdown the expression of LncRNAs (Figure 8A). Then, qPCR and Western Blot were performed to evaluate the regulatory effect of REST. Results showed that both two lncRNAs can reduce the expression levels of REST mRNA (Figure 8B) and protein (Figure 8C). Next, luciferase assay was performed to evaluate the direct binding of miRNA and lncRNA. It was found that miR-129 can directly bind to NR2F2AS1 (Figure 8D), and miR-137 can directly bind to HOTAIRM1 (Figure 8E). Meanwhile, in TCGA-GBM, it can be found that the expression levels of NR2F2AS1 and HOTAIRM1 were significantly higher than that of normal tissues (Figures 8F,G), and the high expression levels were significantly correlated with poor prognosis in TCGA-GBM database (Figures 8H,I). Finally, the mechanistic scheme of lncRNA-miRNA-REST in glioma is shown in Figure 8J.

## Discussion

REST is a member of the Gli-Kruppel family, contains nine Cys2-His2 zinc finger domains, and plays a transcriptional repressive role as a result of the zinc finger domains binding to the RE-1 motif of target genes. Recently, REST has become a hot topic in aging and neurodegenerative research (5, 31, 32). Studies have shown that REST can increase neural excitability and reduce the lifespan *in vivo* (32). In terms of CNS tumors, REST has been confirmed to play key roles in glioma (14–17, 33), although further bioinformatic analysis of distinct roles of REST in glioma remained elusive. The objective of this study was to systematically clarify the clinical relevance, gene functions, and ceRNA regulatory network of REST in glioma.

In the present study, REST expression and clinical data based on the TCGA database was download and analyzed to identify the clinical significance and prognostic value of REST in glioma. High REST expression was positively correlated with higher WHO grade, IDH status (WT), 1p/19q codeletion (non), histological type (GBM), tumor status (with tumor), and primary therapy outcome (SD-PD) (all *p* < 0.001). Moreover, higher REST expression was also significantly related with worse OS, PFI, and DSS in glioma patients, and was a weak independent prognostic factor (*p* = 0.051). These findings indicate an oncogenic role of REST in glioma.

Transcriptional factors (TF) and their regulatory networks are important in tumor development. Therefore, TFs are fast becoming a new direction of antitumor drug discovery (34). It has been suggested that REST may exert its biological effect through histone deacetylation (35), chromatin remodeling (36), methylation (37), and ubiquitination (38). Furthermore, it has been suggested that REST may exert tumorigenic effects by affecting the cell cycle, differentiation, apoptosis (39), proliferation, migration (17), self-renewal (15, 40), and cell signaling pathways, such as Akt-mTOR (10), Hippo (9), TGF-β (41), and Wnt-β-catenin (42). In terms of the functions and pathways of REST DE-mRNAs in glioma, our results showed that BP, such as ECM organization, extracellular structure organization, and neurotransmitter transport; CC, such as collagen-containing ECM, synaptic membrane, and pre-synapse; MF, such as passive transmembrane transporter activity, channel activity, and ECM structural constituents; and pathways, such as neuroactive ligand-receptor interaction and ECM-receptor interaction were remarkably regulated by REST in glioma. Therefore, we analyzed the DE-lncRNA and DE-miRNAs associated with REST expression in gliomas; these DE-ncRNAs may regulate or be regulated by REST either directly or indirectly. Marisetty et al. (43) found that REST regulates oncogenic properties of GBM by repressing miR-124 and miR-203 *in vitro* and *in vivo*, which were in the list that was generated in the current study (Figure 6D); this result partially increases the credibility of our study.

Recently, the ceRNA regulatory mechanism, the novel concept of interactions between ncRNAs and mRNAs, has been used to deeply understand the occurrence and development of cancer (44). Although it is known that REST can be regulated by ncRNAs (7, 45, 46), the upstream regulatory network of REST is still not fully understood. Fortunately, bioinformatics has proven to be an important tool in the study of these regulatory relationships. In the current study, a REST-associated ceRNA network with 4 miRNAs (miR-137, miR-129-1-3p, miR-876-5p, and miR-873-5p) and 9 lncRNAs (NR2F2-AS1, HOTAIRM1, CYTOR, MIR4435-2HG, HAND2-AS1, PVT1, AC009502.1, and AC0098864.1) was successfully constructed based on the TCGA and Starbase. It has been found that mir-137 (47), mir-129-1 (48), and mir-873 (49) exert tumor suppressor functions in glioma, while lncRNAs, including CYTOR (50), HOTAIRM1 (51, 52), MIR4435-2HG (53), and PVT1 (54) maintain tumorigenicity and promote progression of glioma; these studies confirm the reliability of the current findings.

Further, glioma cell lines U251 and T98G were used for subsequent experimental verification. After overexpressing 4 miRNAs using miRNA mimics, we found that only miR-129 and miR-137 could significantly regulate REST at both mRNA and protein levels, and have the ability that inhibits proliferation and migration. Luciferase assays also confirmed that both miR-129 and miR-137 could directly bind to REST 3'UTR. Next, we knocked down their potential regulatory lncRNAs NR2F2-AS1 and HOTAIRM1, respectively, in U251 cells with siRNA. As a result, the knockdown of LncRNAs NR2F2-AS1 and HOTAIRM1 could significantly down-regulate the expression of REST mRNA and protein. Luciferase assays were also confirmed the direct binding of miR-129 to NR2F2-AS1, and miR-137 to HOTAIRM1. Besides, the TCGA-GBM database show that NR2F2-AS1 and HOTAIRM1 are highly expressed and are associated with poor prognosis. Therefore, we finally obtained two REST-related ceRNA regulatory networks in glioma cell lines.

## Conclusion

REST expression was significantly higher in glioma than that in normal samples, and was associated with clinical characteristics. High REST expression was significantly associated with worse OS, worse PFI, and worse DSS in glioma patients. REST may be a prognostic biomarker and potential target of glioma. The REST-associated ceRNA network were constructed based on TCGA database. Finally, REST-associated ceRNA, NR2F2-AS1-miR129-REST and HOTAIRM1-miR137-REST were experimentally validated. Thus, REST may be a prognostic biomarker and target in glioma, and the network validated in this study may provide insights into glioma's molecular regulatory mechanisms.

## Data Availability Statement

The datasets analyzed for this study can be found in The Cancer Genome Atlas database (https://portal.gdc.cancer.gov/), and The Human Protein Atlas (https://www.proteinatlas.org).

## Author Contributions

YZ and YY: conceptualization. YZ, QW, and CZ: methodology. XX, JX, HR, and XS: validation. YZ and QW: formal analysis. YZ, QW, and ZW: experiment. YY: funding acquisition. YZ and ZW: investigation. QW: data curation. YZ: writing—original draft preparation. ZW, LZ, and YY: writing—review and editing. YZ and CZ: visualization. LZ and YY: supervision and project administration. All authors have read and agreed to the published version of the manuscript.

## Funding

This research was funded by the National Natural Science Foundation of China (Grant No. 81872062).

## Conflict of Interest

The authors declare that the research was conducted in the absence of any commercial or financial relationships that could be construed as a potential conflict of interest.

## Publisher's Note

All claims expressed in this article are solely those of the authors and do not necessarily represent those of their affiliated organizations, or those of the publisher, the editors and the reviewers. Any product that may be evaluated in this article, or claim that may be made by its manufacturer, is not guaranteed or endorsed by the publisher.

## Supplementary Material

Supplementary Figure 1 The protein expression of REST in glioma and normal samples using HPA. **(A,B)** The immunohistochemical results of REST protein of two antibodies of glioma and normal tissues were obtained from HPA. **(C)** Statistical column stacking diagram of REST staining in HPA.

Supplementary Table 1 The sequences of the primers or siRNA used in this study. GAPDH and U6 served as endogenous controls for mRNA or miRNA, respectively.

Supplementary Table 2 The list of GO/KEGG enrichment analysis of REST-associated differential expression gene analysis (DEGs).
