# Supplementary figures and images for: Comprehensive Analysis of REST/NRSF Gene in Glioma and Its ceRNA Network Identification
Source: Front Med (Lausanne). 2021 Nov 11;8:739624. doi: 10.3389/fmed.2021.739624 (PMC8631926; doi:10.3389/fmed.2021.739624)

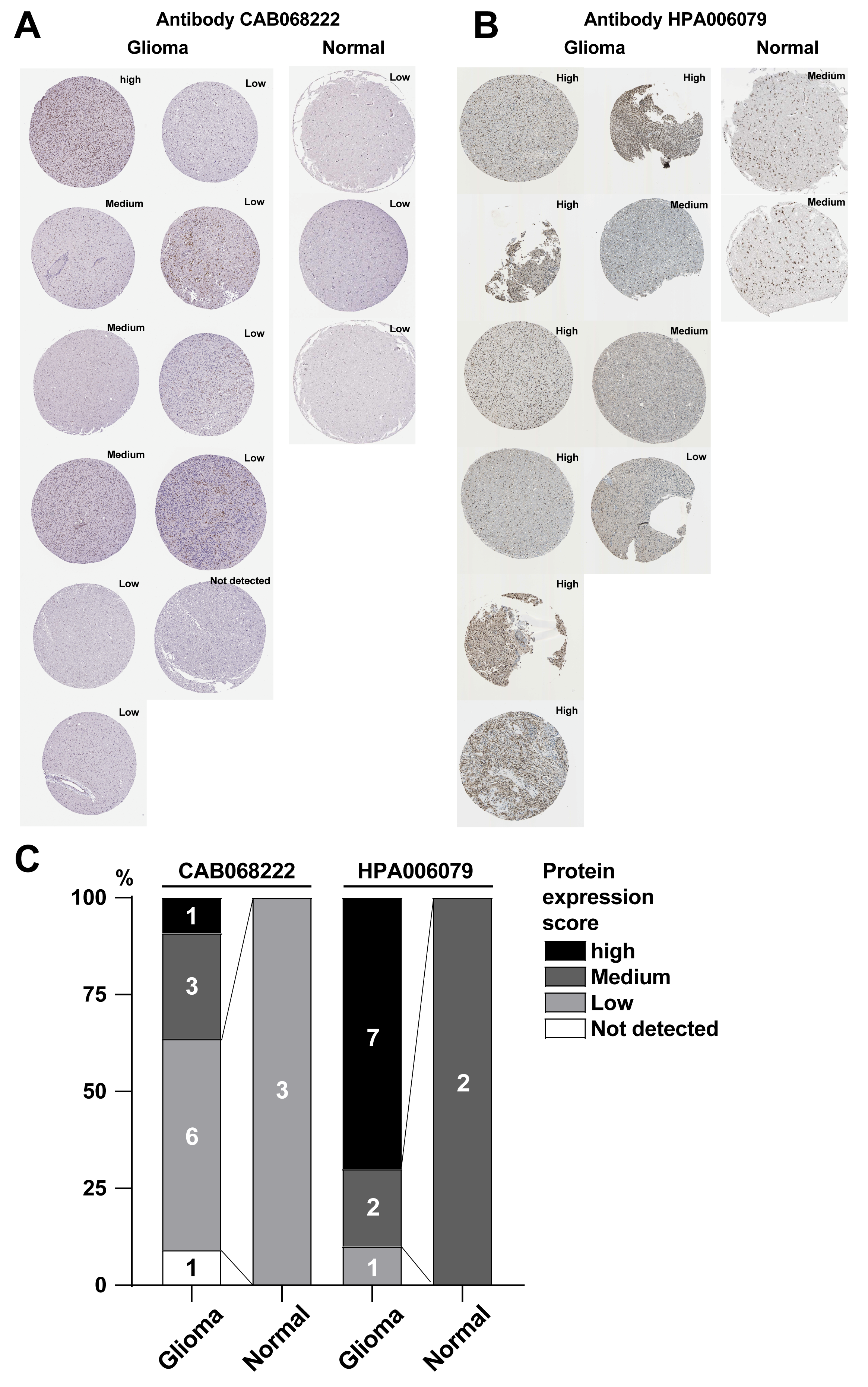

Supplement: Supplementary Figure 1 — The protein expression of REST in glioma and normal samples using HPA. (A,B) The immunohistochemical results of REST protein of two antibodies of glioma and normal tissues were obtained from HPA. (C) Statistical column stacking diagram of REST staining in HPA. [file Image_1.TIF]
