# Supplementary material for: Comprehensive Analysis of REST/NRSF Gene in Glioma and Its ceRNA Network Identification
Source: Front Med (Lausanne). 2021 Nov 11;8:739624. doi: 10.3389/fmed.2021.739624 (PMC8631926; doi:10.3389/fmed.2021.739624)
Supplement: Supplementary Table 1 — The sequences of the primers or siRNA used in this study. GAPDH and U6 served as endogenous controls for mRNA or miRNA, respectively. [file Table_1.docx]

Supplement table 1. The sequences of the primers or siRNA used in this study

| **Sequence Name** | **Sequence 5’一3’** |
| --- | --- |
| GAPDH Forward | GGAGCGAGATCCCTCCAAAAT |
| GAPDH Reverse | GGCTGTTGTCATACTTCTCATGG |
| U6 Forward | GGAACGATACAGAGAAGATTAGC |
| U6 Reverse | TGGAACGCTTCACGAATTTGCG |
| REST Forward | GCCGCACCTCAGCTTATTATG |
| REST Reverse | CCGGCATCAGTTCTGCCAT |
| miR-129-1-3p Forward | CGAAGCCCTTACCCCAAA |
| miR-137 Forward | CGCGCGTTATTGCTTAAGAATAC |
| miR-873-5p Forward | CGCGGCAGGAACTTGTGAG |
| miR-876-5p Forward | CGCGTGGATTTCTTTGTGAA |
| NR2F2-AS1 Forward | TCAGCCGGAAAACTACAAGCTC |
| NR2F2-AS1 Reverse | TCTTCGTGTAGCTGTTCCACC |
| HOTAIRM1 Forward | TTAAATCAACCCGCCCCACA |
| HOTAIRM1 Reverse | TTCAACCCCCTCCCCCATAA |
| si-REST | GCUGGCAAAUGUGGCCUUATT |
| si-NR2F2-AS1 | AAGGATGTCAGCGCACTAAAT |
| si-HOTAIRM1 | AGAAACTCCGTGTTACTCATT |

GAPDH and U6 served as endogenous controls for mRNA or miRNA, respectively.
